# Secondary angle closure glaucoma by lupus choroidopathy as an initial presentation of systemic lupus erythematosus: a case report

**DOI:** 10.1186/s12886-015-0144-6

**Published:** 2015-10-29

**Authors:** Young Soo Han, Chan min Yang, Sang-Hoon Lee, Jae Ho Shin, Sang Woong Moon, Ja Heon Kang

**Affiliations:** Department of Ophthalmology, Graduate School, Kyung Hee University, 26, Kyungheedae-ro, Dongdaemun-gu, Seoul, 02447 Korea; Department of Rheumatology, Kyung Hee University Hospital at Gangdong, Kyung Hee University, 892, Dongnam-ro, Gangdong-gu, Seoul, 05278 Korea; Department of Ophthalmology, Kyung Hee University Hospital at Gangdong, Kyung Hee University, 892, Dongnam-ro, Gangdong-gu, Seoul, 05278 Korea

**Keywords:** Acute angle closure glaucoma, SLE choroidopathy, Ciliochoroidal effusion

## Abstract

**Background:**

We present a rare case of secondary angle closure glaucoma due to systemic lupus erythematosus choroidopathy as initial presentation of systemic lupus erythematosus, accompanied by central nervous system vasculitis and uncontrolled nephropathy.

**Case presentation:**

A 31-year-old woman presented with decreased visual acuity, nausea, vomiting, fever, and bilateral angioedema-like eyelid swelling. She had persistent dry cough while taking medication for 3 months, and had usual posterior neck pain, which was treated with analgesic medication and Asian medicines. Intraocular pressure was 32 and 34 mmHg in her right and left eyes, respectively. Peripheral anterior chambers were shallow (grade I) using the van Herick method. Gonioscopy revealed 360° closed angle in both eyes. In both eyes, serous retinal detachment was found using optical coherence tomography and B scan ultrasonography, as well as choroidal thickening with effusion. Secondary acute angle closure glaucoma was drug induced, or caused by uveitis of unknown etiology when she was first treated with intraocular pressure-lowering medication. During evaluation of the drug-induced angioedema in the internal medicine department, systemic lupus erythematosus was diagnosed, based on malar rash, photosensitivity, proteinuria, and positive anti-Smith and anti-DNA antibodies, followed by initiation of steroid pulse therapy. Using fluorescein angiography, multifocal subretinal pinpoint foci were detected at the middle phase. We then diagnosed bilateral angle closure glaucoma by choroidal effusions, with lupus choroidopathy. At 2 months after steroid pulse therapy, subretinal fluid was not found, and visual acuity improved to normal. During the subsequent 2 years, lupus choroidopathy was not aggravated but lupus nephritis was not controlled.

**Conclusion:**

Patients with systemic lupus erythematosus choroidopathy can develop ciliochoroidal effusion, which can lead to acute angle closure glaucoma. Systemic lupus erythematosus choroidopathy is an early sign of severe complications. Angle closure glaucoma by systemic lupus erythematosus choroidopathy can be effectively treated using antiglaucoma drugs and immunosuppressive therapy.

## Background

Systemic lupus erythematosus (SLE) is a chronic autoimmune multisystem disease [1]. The diagnosis is based on the American College of Rheumatology criteria (1982), if four or more of the manifestations are presented, either serially or simultaneously during any interval of observation [2]. SLE may affect joints, derma, kidneys, and the brain. Clinical manifestations vary from mild joint and dermal problems to severe outcomes, including renal, cardiologic, and neurologic disorders. SLE may affect almost every part of the eye. Blepharitis, keratoconjunctivitis sicca, scleritis, retinal vascular disease, and neuro-ophthalmic diseases are typical manifestations [3]. Unlike these clinical manifestations, choroidopathy is a rare complication, with only 28 cases reported since 1968 [4]. Furthermore, accurate angle closure glaucoma associated with choroidopathy in SLE was reported in only two cases [5, 6]. However, these previous two cases reported relatively mild nephritis and no central nervous system (CNS) disorders. In the following report, we therefore describe a case of bilateral angle closure glaucoma with lupus choroidopathy at initial presentation of SLE, with severe systemic complications of CNS vasculitis and uncontrolled nephritis.

## Case presentation

A 31-year old Korean woman presented with a 4-day history of decreased bilateral visual acuity, accompanied by nausea, vomiting, fever, and bilateral angioedema-like eyelid swelling. She had persistent dry cough after being treated with medication for 3 months. She also had usual posterior neck pain, so a brain magnetic resonance imaging was taken 10 days later, but the results were normal and treated with analgesic medication and Asian medicines. There was no significant ocular disease history or family history.

**Fig. 1:**
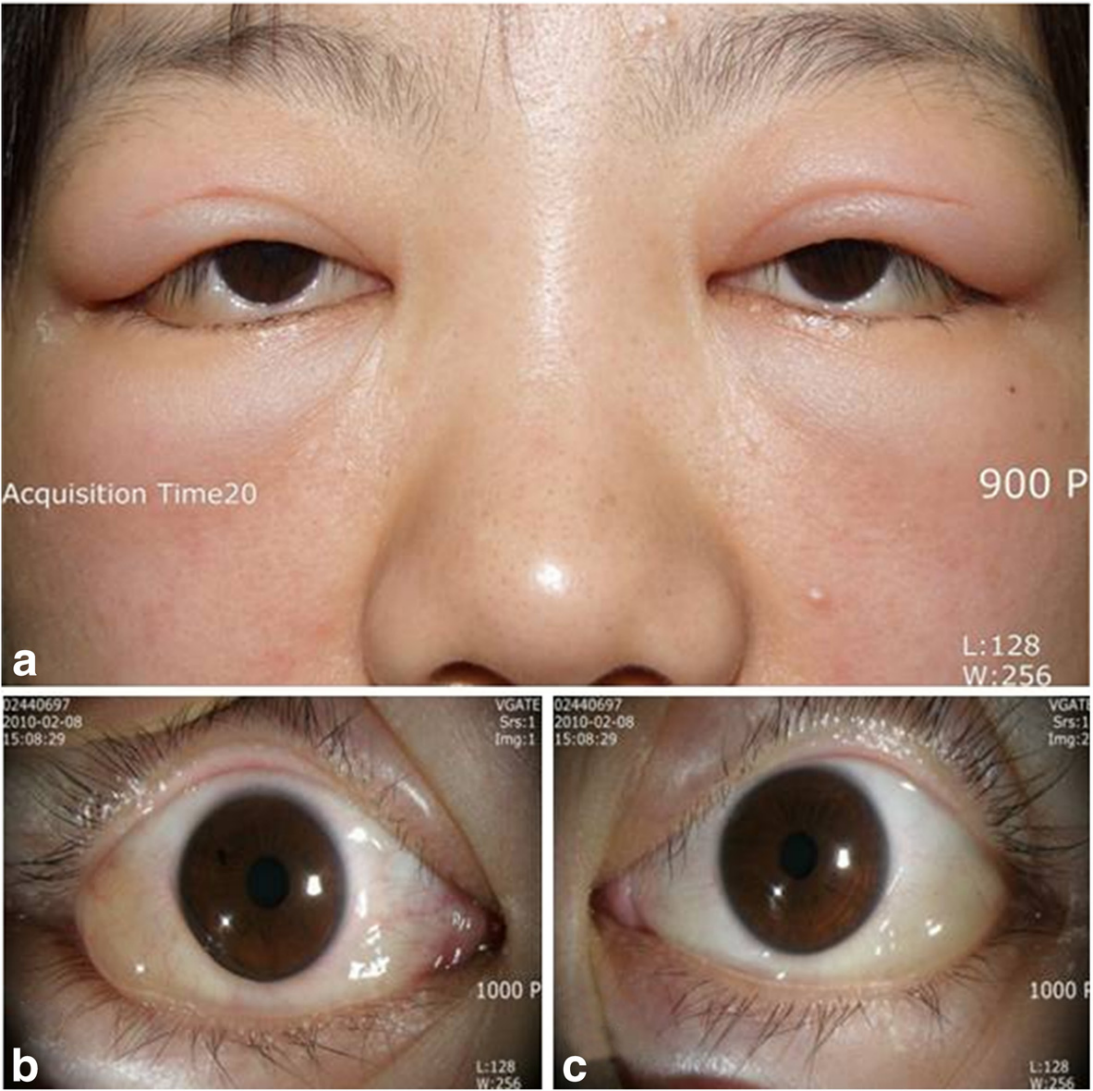
Anterior segment photographs of both eyes. Eyelid edema (**a**) and chemosis (**b**, **c**) were found in both eyes

**Fig. 2:**
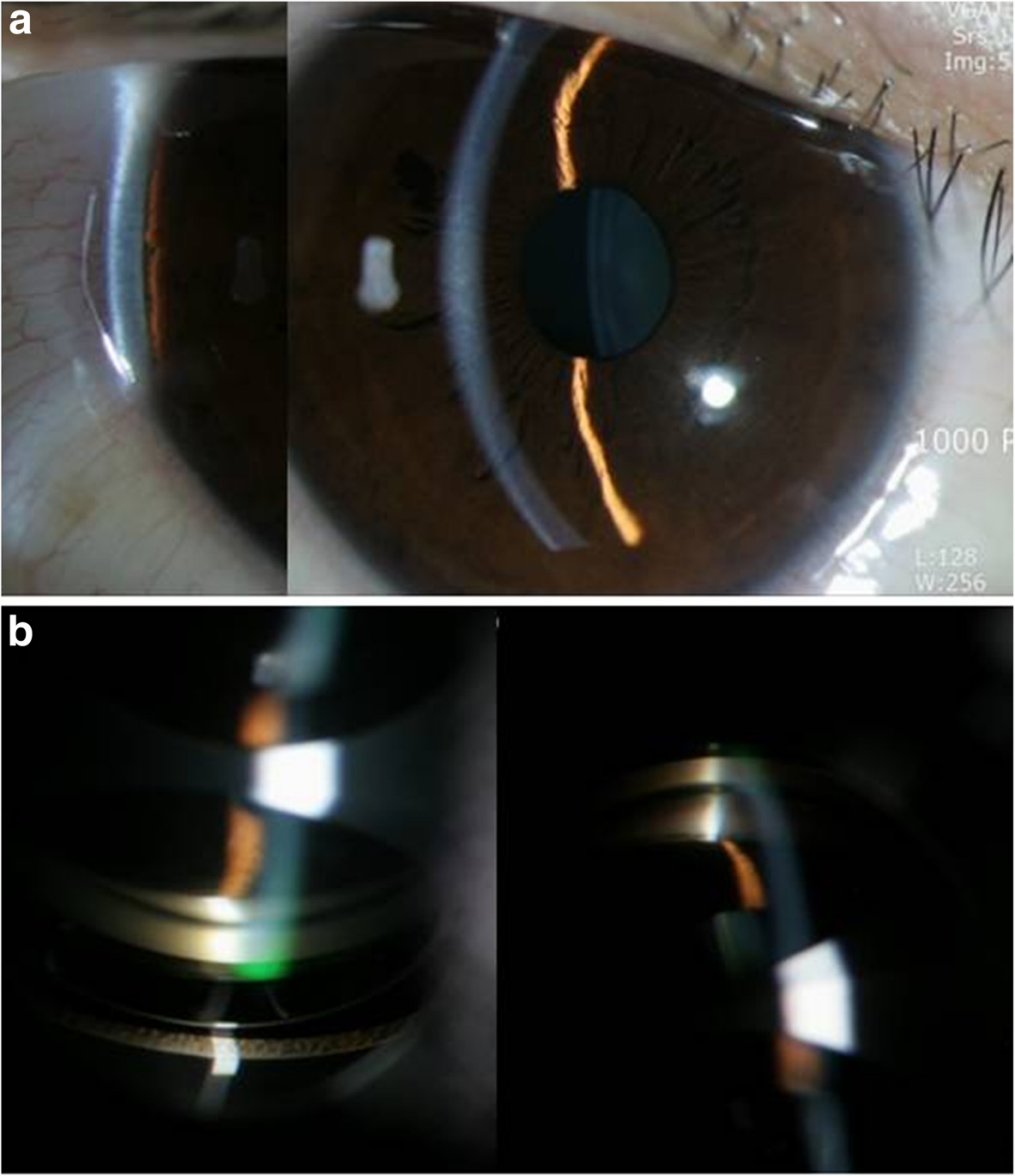
Slit lamp findings (**a**) and gonioscopy (**b**). Anterior chamber was grade 1 using the van Herick method (**a**), 360° occlusion was found with gonioscopy (**b**)

**Fig. 3:**
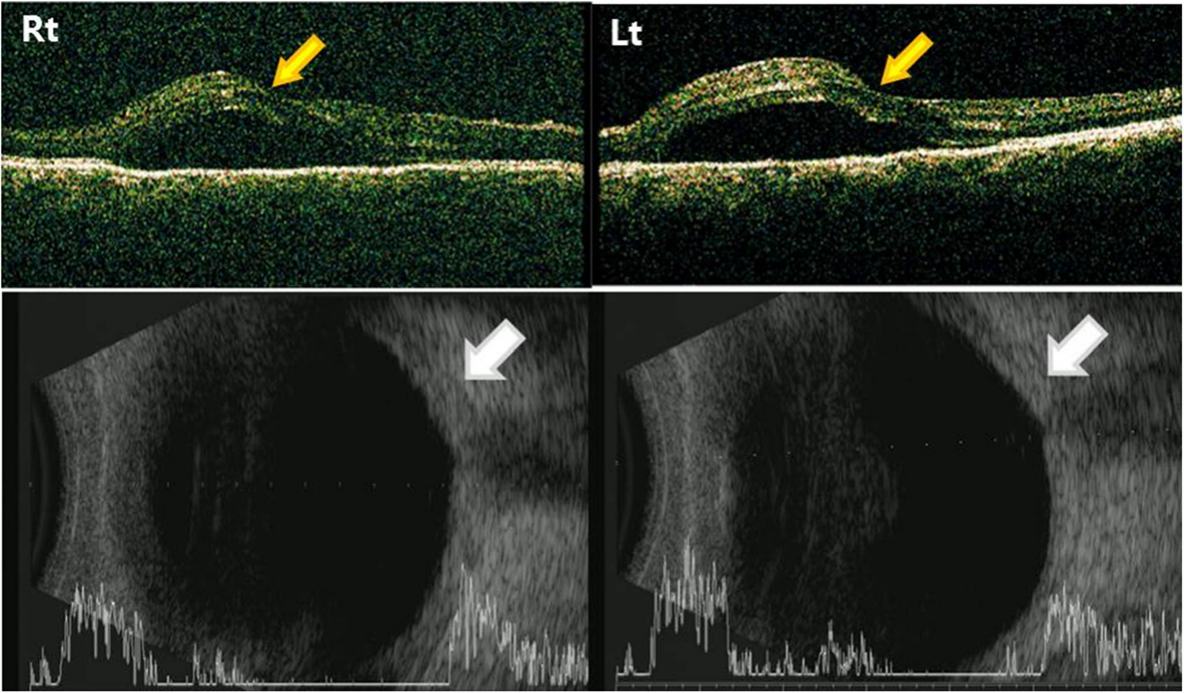
Optical coherence tomography (OCT) and B scan ultrasonography of both eyes. Choroidal thickening with effusion (white arrow) were found in both eyes. Using OCT, serous retinal detachment (yellow arrow) was found in both eyes

On ophthalmic examination, best corrected visual acuity was 20/32 in the right eye and 20/40 in the left eye, with −5.00 spherical equivalent in both eyes. The intraocular pressure (IOP) was 32 mmHg in the right eye and 34 mmHg in left eye, using a Goldman applanation tonometer. She also had bilateral edema-like angioedema in the eyelids, and facial and severe chemosis (Fig. 1). Slit lamp biomicroscopy revealed clear corneas in both eyes. Using the van Herick method, peripheral anterior chambers were shallow and classified as grade I, but chamber reactions were not found. Gonioscopy revealed 360° closed angle in both eyes (Fig. 2). Using optical coherence tomography (OCT) and B scan ultrasonography, serous retinal detachment was detected in both eyes, in addition to choroidal thickening with effusion (Fig. 3). Initially, we diagnosed secondary angle closure glaucoma due to drug induction or to uveitis of unknown etiology. We treated the patient with IOP-lowering medications (topical dorzolamide and timolol twice a day in both eyes, plus oral acetazolamide twice a day), and planned to consult with the internal medicine department concerning the angioedema and uveal effusion.

During evaluation of the drug-induced angioedema by the internal medicine department, a diagnosis of SLE was made, based on malar rash, photosensitivity, proteinuria (24-h protein, 682 mg), and positive anti-Smith and anti-DNA antibodies. She was given intravenous methylprednisolone, 250 mg per day. 9 days after treatment, the IOP decreased to 21 mmHg in both eyes. Periorbital swelling and conjunctival chemosis decreased, and symptoms of nausea decreased. She was discharged with a prescription of 20 mg of prednisolone, to be taken each day.

**Fig. 4:**
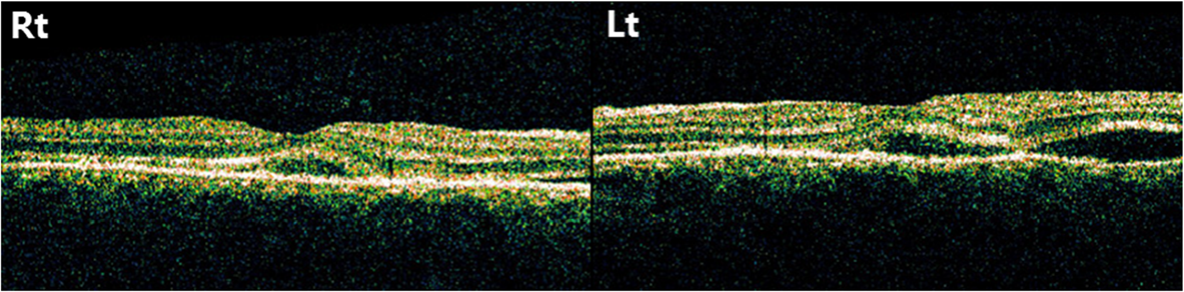
OCT of both eyes. Serous retinal detachment was decreased more than Fig. 3 in both eyes

**Fig. 5:**
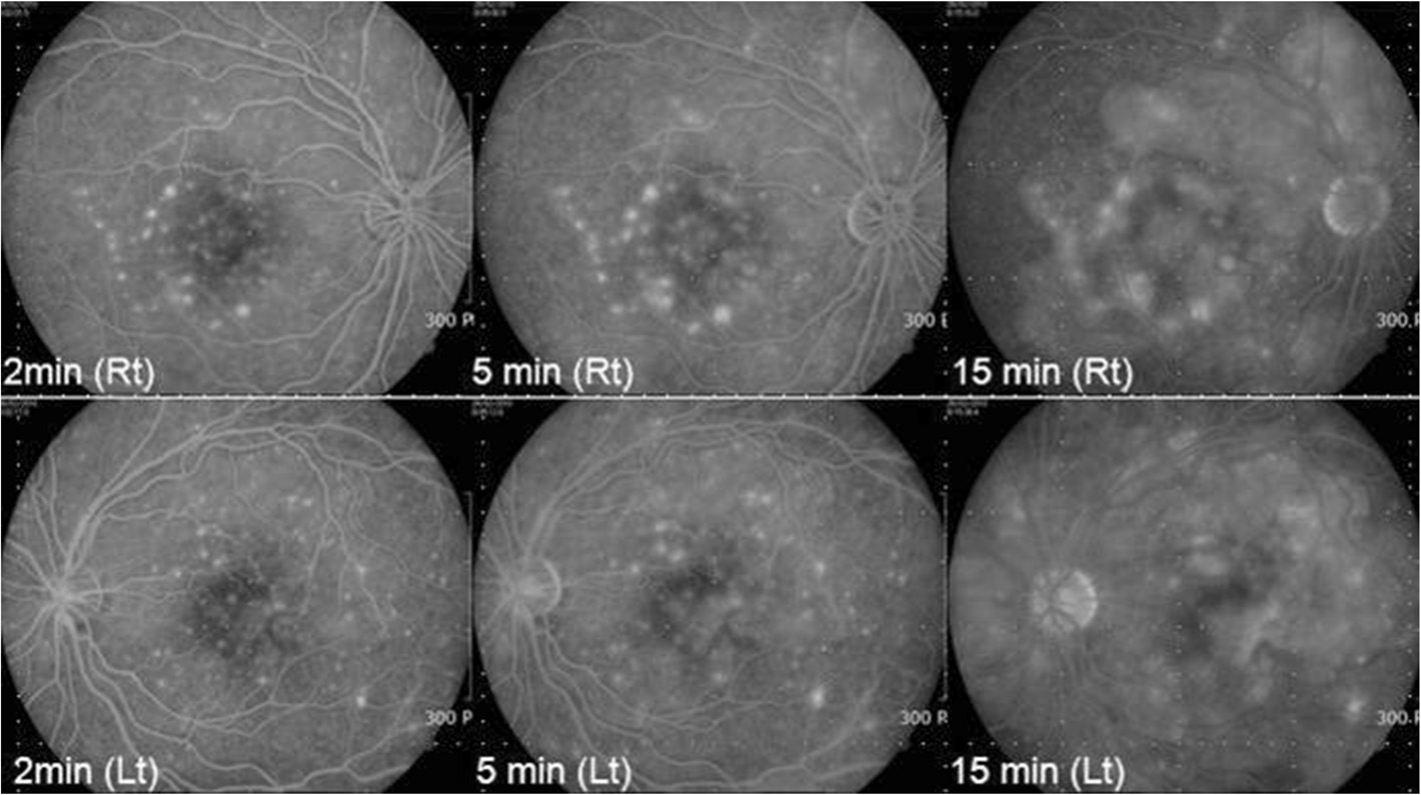
Fluorescein angiography (FAG) at the initial visit. Multifocal subretinal pinpoint foci were detected at the middle phase, and serous elevation was detected at the late phase in both eyes

She returned 5 days later with increasing periorbital swelling, severe conjunctival chemosis, orthopnea, coughing, and headache. Chest computed tomography revealed bilateral pleural effusion and atelectasis. Cardiac sonography showed pericardial effusion. Using a brain MRI, SLE vasculitis was found in the thalamus and basal ganglia. She was treated with thoracocentesis that showed exudates, and also treated with high doses of intravenous methylprednisolone (1 g). At this time, IOP in both eyes was 18 mmHg with medication, and the peripheral anterior chamber was deepened. Using OCT, decreased serous retinal detachment was found in both eyes (Fig. 4). Using fluorescein angiography (FAG), multifocal subretinal pinpoint foci were detected at the middle phase, and serous elevation was detected at the late phase (Fig. 5). The diagnosis was changed to secondary angle closure glaucoma by choroidal effusion with lupus choroidopathy. After steroid pulse therapy, the patient’s systemic symptom was relieved, so she was discharged with a prescription of 60 mg prednisolone and 200 mg hydroxychloroquine.

**Fig. 6:**
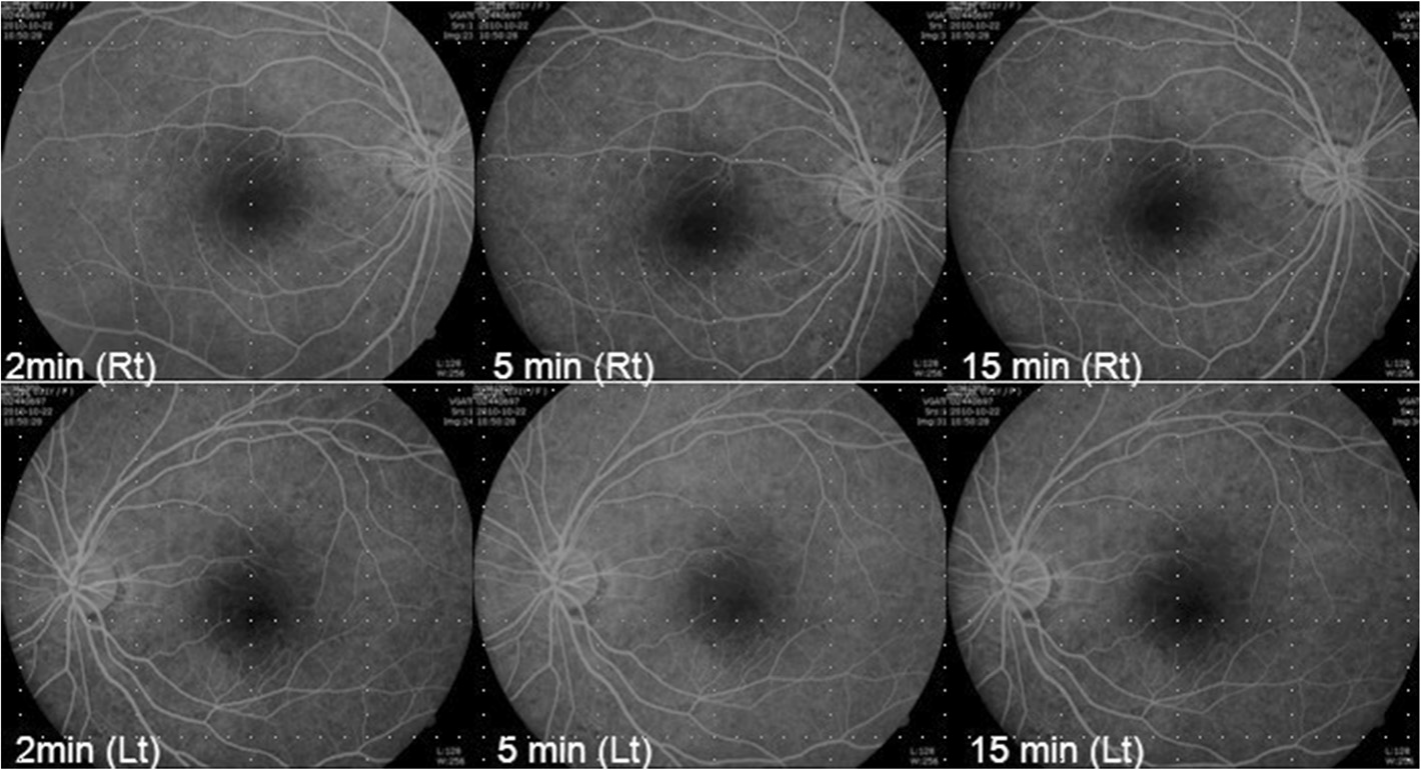
FAG 8 months after the initial visit. Subretinal leakage was not found in either eye

One month after the first visit, her best corrected visual acuity was 20/32 in both eyes, with −2.75D for the right eye and −3.00D for the left eye. After medication, IOP was 17 mmHg in both eyes. FAG showed no leakage from choroidal vessels (Fig. 6). Peripheral anterior chambers deepened, but OCT detected some subretinal fluid, which was not as severe as before treatment. Two months after the first visit, OCT detected no subretinal fluid, and visual acuity improved to 20/20 in both eyes, with IOP well controlled. Lupus choroidopathy was in remission, with no aggravation for 2 years, but systemic problems such as nephritis were not controlled. Although various medications such as steroids, azathioprine, mycophenolate mofeti, and cyclophosphamide were given, nephritis was still not controlled and was aggravated for 2 years.

## Discussion

Although SLE is a chronic idiopathic autoimmune disease caused by autoantibodies and complement fixing immune complexes associated with an abnormally activated immune system, the exact pathologic mechanisms are unknown [7]. Our patient presented with malar rash, serositis (pleural effusion, pericardial effusion), renal disorder (proteinuria greater than 0.5 g per day), neurologic disorders (headache and CNS vasculitis), immunological disorders (anti-DNA antibodies), and positive antinuclear antibody (ANA) (titer 1280:1, speckled). These six criteria were consistent with a diagnosis of SLE.

SLE may affect almost any part of the eye. Ocular manifestations include blepharitis, keratoconjunctivitis sicca, scleritis, retinal vascular disease, and neuro-ophthalmical diseases. SLE choroidopathy is a rare disease, and is associated with severe complications that can involve nephritis and CNS disorders [8].

SLE choroidopathy may present as multiple serous detachments of the retinal pigment epithelium and the neurosensory retina. FAG and indocyanine green antiography (ICGA) are very helpful for diagnosis. FAG typically detects multifocal round serous detachments of the retina and/or retinal pigment epithelium (RPE). It also detects retinal vessel changes, and indirectly suggests choroid involvement [8]. Though we did not perform ICGA, this methodology can detect choroidal abnormalities that are not detectable using FAG and fundus examination. At the early phase, focal areas of choroidal ICGA hypofluorescence indicate choroidal filling delays. At the late phase, late diffuse zonal choroidal ICGA hyperfluorescence may indicate choroidal vascular leakage, probably caused by choroidal vessel wall damage. At intermediate to late phases, focal clusters of pinpoint spots of ICGA choroidal hyperfluorescence indicate ICGA staining, with abnormal focal fixing of the ICG molecule. Transient early hypofluorescence and late hyperfluorescence are also observed in other inflammatory and non-inflammatory ocular diseases, but intermediate to late, focal clusters of pinpoint spots of ICGA choroidal hyperfluorescence are a unique finding. The focal clusters of pinpoint spots of ICGA choroidal hyperfluorescence may represent immune deposits at the choroidal stroma, Bruch’s membrane, or RPE basement membrane [9]. Because ICGA is more sensitive than FAG for SLE choroidopathy, ICGA is essential for diagnosis of this disorder.

A differential diagnosis of SLE choroidopathy includes Vogt–Koyanagi–Harada syndrome, central serous chorioretinopathy (CSC), and hypertensive choroidopathy. Especially for SLE patients who previously receive steroid therapy, CSC is very difficult to distinguish from SLE choroidopathy. SLE choroidopathy is bilateral and has multiple leakage points, but CSC is usually unilateral (more than 60 %) [10], and has one or two leakage points located within 500–1500 μm of the center of the fovea [11]. In our case, multiple leakage points were found in FAG findings. The patient did not receive steroid therapy, so we excluded CSC. Hypertensive choroidopathy was also excluded because our patient was normotensive, and was not matched to the Vogt–Koyanagi–Harada syndrome diagnostic criteria [12].

The pathogenesis of choroidopathy remains unclear, but it is thought to involve three factors. First, it may involve immune complex deposition at the choroid and choriocapillaris, and the presence of autoantibodies against RPE [13], that may lead to hypoperfusion and secondary breakdown of the blood–retinal barrier. Second, uncontrolled hypertension caused by SLE nephropathy may constrict choroidal blood flow, with resultant ischemia and breakdown of the outer blood–retinal barrier at the RPE [14]. Third, thrombosis can also contribute to choroidopathy by causing microangiopathy [13]. A combination of these three factors creates hypoperfusion of the choriocapillaris, resulting in RPE damage and leakage of liquid into the subretinal space.

SLE choroidopathy generally occurs in patients who have highly active diseases, like our patient who had CNS vasculitis and nephropathy. Nguyen et al. [13] reported that all SLE choroidopathy patients had active SLE. Of these patients, 64 % had nephropathy and 36 % had CNS involvement. In our patient, CNS involvement was mild, but nephropathy was not controlled after 2 years, despite treatment with many types of immunosuppressive drugs. The cause of the disorder involving both the choroid and kidney involves similar pathogenesis and structures. Both organs are highly susceptible to immune complex deposition that leads to fluid displacement into the interstitium [15]. Additionally, the Bruch’s-RPE choriocapillary membrane has a morphological resemblance to the glomerulus of kidney. Choriocapillary is similar to the glomerular capillary tuft, Bruch’s membrane is similar to the glomerular basement membrane, and the RPE is similar to the glomerular epithelium [8].

Bilateral angle closure glaucoma involves ciliochoroidal effusion associated with SLE choroidopathy. Ciliochoroidal effusion is thought to involve transudative fluid escape from the choriocapillaris into the surrounding potential space (suprachoroidal space), causing edema and choroidal detachment [16]. As previously mentioned, RPE damage in SLE choroidopathy facilitates leakage of protein into the suprachoidal space, increasing osmotic pressure [5]. Furthermore, a systemic hyperosmolar state from proteinuria and hypoalbuminemia associated with nephrotic syndrome contributes to the leakage of fluid into the suprachoroidal space [6]. Ciliary body edema can induce anterior movement of the lens-iris-diaphragm, resulting in a shallow anterior chamber because of an anterior rotation of the ciliary body around the scleral spur. Ciliary body edema relaxes the lens zonule, leading to a thickening of the crystallin lens and a myopic shift [17]. In our case, B scan ultrasonography revealed choroidal effusion and about a three diopter myopic shift (initial visit: −5.00 diopter, resolved state: −2.00 diopter).

To treat angle closure glaucoma due to SLE choroidopathy, systemic immunosuppressive therapy should first be used. High doses of corticosteroids and addition of cyclophosphamide have been effective for choroidopathy. As previously mentioned, SLE choroidopathy is associated with nephropathy and CNS vasculitis. A previous study reported that four of 28 patients with choroidopathy died from lupus-related complications [18]; thus, prompt and aggressive immunosuppressive therapy should be used. Hypertension control, that involves diuretic therapy contributing to the development of choroidopathy, may be effective. If immunosuppressive therapy is not effective for choroidopathy, focal laser treatment at the point of choroidal leakage may result in the resolution of serous retinal detachment and restoration of visual acuity [19]. To control IOP, topical ocular hypertensive agents and topical cycloplegics are recommended. In conventional angle closure glaucoma, topical cycloplegics are contraindicated, and in ciliary body edema, topical cycloplegics decrease IOP by inducing a posterior rotation of the ciliary body [17]. After medical therapy fails to control glaucoma, peripheral iridectomy and surgical treatment with choroidal drainage may also be considered [6].

## Conclusions

We report bilateral angle closure glaucoma due to SLE choroidopathy as initial presentation of SLE. This case could have been misdiagnosed as conventional angle closure glaucoma. Ciliochoroidal effusion could be detected by B scan ultrasonography, and ultrasound biomicroscopy, and ICGA is especially helpful in the diagnosis of SLE choroidopathy. This case is typical of SLE choroidopathy, which is often accompanied by severe SLE complications such as uncontrolled CNS vasculitis and nephropathy. Prompt and aggressive immunosuppressive therapy is therefore recommended.

## Consent

Written informed consent was obtained from the patient for publication of this Case Report and the accompanying images. A copy of the written consent is available for review by the Editor of the journal.

## Abbreviations

SLE: Systemic lupus erythematosus
CNS: Central nervous system
IOP: Intraocular pressure
OCT: Optical coherence tomography
FAG: Fluorescein angiography
ICGA: Indocyanine green angiography
RPE: Retinal pigment epithelium
CSC: Central serous chorioretinopathy
